# Predicting Future High-Cost Schizophrenia Patients Using High-Dimensional Administrative Data

**DOI:** 10.3389/fpsyt.2017.00114

**Published:** 2017-06-30

**Authors:** Yajuan Wang, Vijay Iyengar, Jianying Hu, David Kho, Erin Falconer, John P. Docherty, Gigi Y. Yuen

**Affiliations:** ^1^Innovation and Foundational Technology, IBM Watson Health, Yorktown Heights, NY, United States; ^2^IBM T.J. Watson Research Center, Yorktown Heights, NY, United States; ^3^Medical Strategy, ODH, Inc., Princeton, NJ, United States

**Keywords:** health-care cost, machine learning, feature selection, model selection, schizophrenia

## Abstract

**Background:**

The burden of serious and persistent mental illness such as schizophrenia is substantial and requires health-care organizations to have adequate risk adjustment models to effectively allocate their resources to managing patients who are at the greatest risk. Currently available models underestimate health-care costs for those with mental or behavioral health conditions.

**Objectives:**

The study aimed to develop and evaluate predictive models for identification of future high-cost schizophrenia patients using advanced supervised machine learning methods.

**Methods:**

This was a retrospective study using a payer administrative database. The study cohort consisted of 97,862 patients diagnosed with schizophrenia (ICD9 code 295.*) from January 2009 to June 2014. Training (*n* = 34,510) and study evaluation (*n* = 30,077) cohorts were derived based on 12-month observation and prediction windows (PWs). The target was average total cost/patient/month in the PW. Three models (baseline, intermediate, final) were developed to assess the value of different variable categories for cost prediction (demographics, coverage, cost, health-care utilization, antipsychotic medication usage, and clinical conditions). Scalable orthogonal regression, significant attribute selection in high dimensions method, and random forests regression were used to develop the models. The trained models were assessed in the evaluation cohort using the regression *R*^2^, patient classification accuracy (PCA), and cost accuracy (CA). The model performance was compared to the Centers for Medicare & Medicaid Services Hierarchical Condition Categories (CMS-HCC) model.

**Results:**

At top 10% cost cutoff, the final model achieved 0.23 *R*^2^, 43% PCA, and 63% CA; in contrast, the CMS-HCC model achieved 0.09 *R*^2^, 27% PCA with 45% CA. The final model and the CMS-HCC model identified 33 and 22%, respectively, of total cost at the top 10% cost cutoff.

**Conclusion:**

Using advanced feature selection leveraging detailed health care, medication utilization features, and supervised machine learning methods improved the ability to predict and identify future high-cost patients with schizophrenia when compared with the CMS-HCC model.

## Introduction

Schizophrenia is a chronic and costly condition estimated to have annual direct and indirect costs of up to US$102 billion (1, 2). Individuals with schizophrenia often suffer repeated emergency department (ED) visits and hospitalizations and require complex care management strategies for comorbid medical conditions. Given the significant cost and disease burden of serious and persistent mental illness, health-care organizations require adequate risk adjustment models to effectively allocate their resources to managing patients who are at the greatest risk.

Despite a growing interest by advocacy groups and US policymakers in measuring outcomes and managing mental health care, risk adjustment specific for mental health conditions has received little attention in commercial risk-adjustment models (3). Health-care organizations have historically used diagnosis-based and data-driven risk adjustment models that cover general medical conditions. These models include the Hierarchical Condition Categories (HCCs) and the Adjusted Clinical Groups (ACGs) systems. The ACG model predicts health-care resource utilization based on the presence or absence of specific aggregated diagnosis groups, recorded on inpatient and outpatient service claims over a given time period (e.g., a year), as well as age and gender, to classify individuals into ACG categories. The HCC model, developed by the Centers for Medicare & Medicaid Services (CMS) in 2004, also uses diagnostic codes and demographic data aggregated into condition categories, and assigns each patient a single risk score (4, 5).

Both ACG and Centers for Medicare & Medicaid Services Hierarchical Condition Categories (CMS-HCC) models have fair predictive value for predicting hospitalizations (6, 7). However, in general, these models have been found to underestimate total health-care costs for individuals with mental or behavioral health conditions and overestimate the costs for those without these issues (3, 8, 9). In behavioral health populations, these models have been shown to explain no more than 10% of the variance in mental health and substance abuse spending (3, 8, 9). Adding more precise behavioral health (mental health and substance use) diagnoses to the commercial risk adjustment models has been shown to improve their ability to predict health-care costs (10).

In the current study, we evaluate the predictive power of using advanced supervised machine learning methods to identify and predict future high-cost schizophrenia patients. We used a hybrid approach to identifying risk factors to optimize prediction by starting with risk factors driven by knowledge (i.e., patient characteristics hypothesized to predict risk) and augmenting with additional risk factors extracted from the data. Utilizing a dataset from claims, enrollment, and coverage data sources, we aimed to develop predictive models for the identification of future high-cost patients with schizophrenia and compare the performance of the models with the current CMS-HCC model.

## Materials and Methods

### Data Overview

A retrospective study was conducted to develop and evaluate a predictive model for identifying future high-cost patients with schizophrenia. Payer administrative data were obtained from a third party, a multi-state, multi-insurer commercial insurance database, which contains claims, benefit enrollment, and coverage information from 97,862 patients who had at least one schizophrenia ICD9 code (295.*) between January 2009 and June 2014. The medical/pharmacy claims contain diagnosis, procedure, and drug information as well as provider specialty, place of service, and claim amounts. The enrollment and coverage data contain patient demographic information, product type, pay type, and the associated coverage periods.

### Study Design

**Figure 1:**
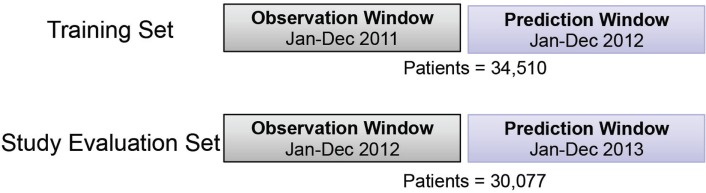
Illustration of training and study evaluation cohorts for the study design.

In this study, the prediction model of health expense of schizophrenia patients in the upcoming year was developed and evaluated. The corresponding training and evaluation cohorts were derived based on calendar year. Twelve months were used as an observation window (OW); and the following 12 months were used as a prediction window (PW). The study-eligible population included all patients with commercial or self-insured plans, full medical and pharmacy coverage under the same product and pay type in the OW; and with at least January coverage in the following PW. A total of 34,510 patients were included in the training cohort with 2011 OW and 2012 PW; similarly, 30,077 patients were included in study evaluation cohort with 2012 OW and 2013 PW (Figure 1). To identify future high-cost schizophrenia patients, the average per member per month (PMPM) of total cost in the PW was used as the regression target of predictive modeling in this study. Different top percentiles of total cost PMPM were selected to stratify patients into high- vs low-cost groups.

### Designed Features

Table 1 shows the six feature types and examples designed and investigated in this study (~70,000 features), including demographics, coverage, cost, health-care utilization, antipsychotic medication usage, and clinical condition proxies. Demographic features included age, gender, and geographic region based on residence (e.g., Northeast, South, Midwest, West). Coverage features included payer type (e.g., commercial) and product type (e.g., preferred provider organization, health maintenance organization) in the OW and separately in January of the PW.

**Table 1:** Variables investigated in the three models studied.

Variable category	Variable examples (descriptions)	No. of variables	Baseline model	Enhanced model	Final model
Demographics	Age, gender, region, etc.	8	X	X	X

Coverage	Payer type, product type (e.g., PPO, HMO, etc.) in the observation window and January prediction window	32		X	X

Cost	12-month PMPM for total cost 12-month detail costs (12-month average PMPM for inpatient, outpatient, ED visit, office visit, and Rx) Recent detail costs (11) (most recent 6- and 3-month average PMPM for total, inpatient, outpatient, ED visit, office visit, and Rx) Cost pattern proxies (number of months with cost above mean; max monthly cost, etc.)	1 5 12 2	X	X X X X	X X X X

Health-care utilization	Counts of ED visit, office visit, hospitalization; normalized number of hospitalization days	4		X	X

Antipsychotic medication usage	Medication adherence—cumulative possession ratio (12, 13) Generation switch between the first and second generation of antipsychotic drugs; count of primary compounds in prescription	1 4		X X	X X

Clinical condition proxies	Counts of ICD-9 codes in 12 months, in the first 6 months only, in the last 6 months only; counts of ICD-9 grouping in 12 months, counts of procedure code in 12 months; most recent inpatient stay’s primary ICD-9 code and discharge status The count of total number of days for different prescriptions by generic product identifier and by generic name in 12 months The indicator of clinical condition in 12 months (CMS condition categories and CMS hierarchical condition categories)	68,654 2,639 140			X X X

*CMS, Centers for Medicare & Medicaid Services; ED, emergency department; HMO, Health Maintenance Organization; ICD, International Classification of Diseases; PMPM, per member per month; PPO, Preferred Provider Organization; Rx, prescription*.

Cost variables in the OW give a global picture of patients’ health status and are critical for future cost forecast. Costs were characterized based on standard utilization buckets—inpatient, outpatient, ED, physician office visit, and Rx services. Different PMPMs were extracted from individual claims as normalized target variables of costs. Costs in the second half year as well as the fourth quarter of OW in each utilization bucket were extracted to capture the individual expense trajectory. Furthermore, specific variables were generated to capture whether a patient’s cost exhibits a “spike” pattern (11), i.e., a sudden rising and dropping cost. This cost pattern indicator was represented by the number of months with claim cost above the PMPM in 12-month OW. For example, if an individual has only 1 month cost above PMPM (i.e., “spike” cost), it suggests an acute/accident cost; whereas the presence of multiple months with spike costs indicates a typical cost pattern for chronic conditions. Different cost patterns can lead to different probabilities of cost recurrence. We also used the maximum monthly cost in the OW.

In addition to cost, individual health-care utilization was also captured by the number of services in the OW, such as ED visits, physician office visits, hospitalizations, as well as length of hospital stay. Antipsychotic medication usage was derived, including cumulative possession ratio for medication adherence (12, 13), different generations of antipsychotic drugs prescribed to patients as a proxy for “side effect mitigation,” as well as the counts of primary compounds in the prescription as a proxy for “medication complexity.”

Patients’ clinical/diagnostic conditions were inferred from ICD-9 codes, procedure codes, and prescriptions associated with claims. Given the large number of different codes and drug names, a specific code or drug name can show up infrequently in the whole cohort; therefore, we refer to those variables with infrequent occurrences as sparse features. The complexity of individual’s comorbidities was captured on different levels: (1) count of individual ICD-9 codes; (2) count of ICD-9 groups based on the coding structure (e.g., 250.00–250.93 belong to group 250); (3) count of condition categories developed by CMS (CMS-CC) and hierarchical condition categories (CMS-HCC) (14). To capture the trajectory of clinical conditions, distinct ICD-9 codes were counted only in the first half or only in the second half of the OW. Furthermore, for individuals who had hospitalizations, the primary ICD-9 code associated with the latest hospitalization was extracted, as well as the discharge status, both of which have a strong tendency to indicate the future health-related expense. The complexity of treatment and intervention was derived by counting the different procedure codes in the OW; complexity of medication status was derived by counting: (1) the drug classes of Generic Product Identifier and (2) generic names from pharmacy claims in the OW.

### Predictive Modeling

To assess the value of different variable categories for health expense forecast, the predictive models were developed and evaluated in three stages: (1) *Baseline Model* using only demographic (age, gender) and total cost features in OW; (2) *Enhanced Model* adding coverage, health-care utilization, antipsychotic medication usage, and detailed costs to the Baseline Model; (3) *Final Model* adding sparse features to the Enhanced Model (Table 1). 10-fold cross validation on the training cohort was utilized to obtain training performance. The trained models were evaluated in 30,077 study patients to establish testing performance.

Two in-house sparse feature selection methods were investigated for the development of the Enhanced Model, and especially for the Final Model. Scalable Orthogonal Regression feature selection has the advantage to expand a set of knowledge-driven risk factors with additional data-driven variables (15). The method is designed to select less redundant features without sacrificing the prediction power, for which redundancy is measured by an orthogonality measure added as a penalty term in the regression cost function. Attributes were also selected using the Likelihood Ratio Test based on the normal distribution. Following the methodology in Kulldorff et al. (16), Monte Carlo experiments with permuted targets were performed to compensate for extensive multiple testing of a large number of potential attributes. A variety of regression algorithms, including linear regression, decision tree regression, and random forests regression, were investigated for the model development. An ensemble method of Random Forests Regression was chosen given the capability to capture non-linear interaction in feature variables as well as the different sub-cohorts in the heterogeneous population in the Final Model. Model performances were also compared to the industry standard CMS-HCC 2013 Risk Adjustment Model. This comparison should be made with the awareness that our model represents an extrapolation of the CMS-HCC model that was originally developed based on the Medicare population.

**Figure 2:**
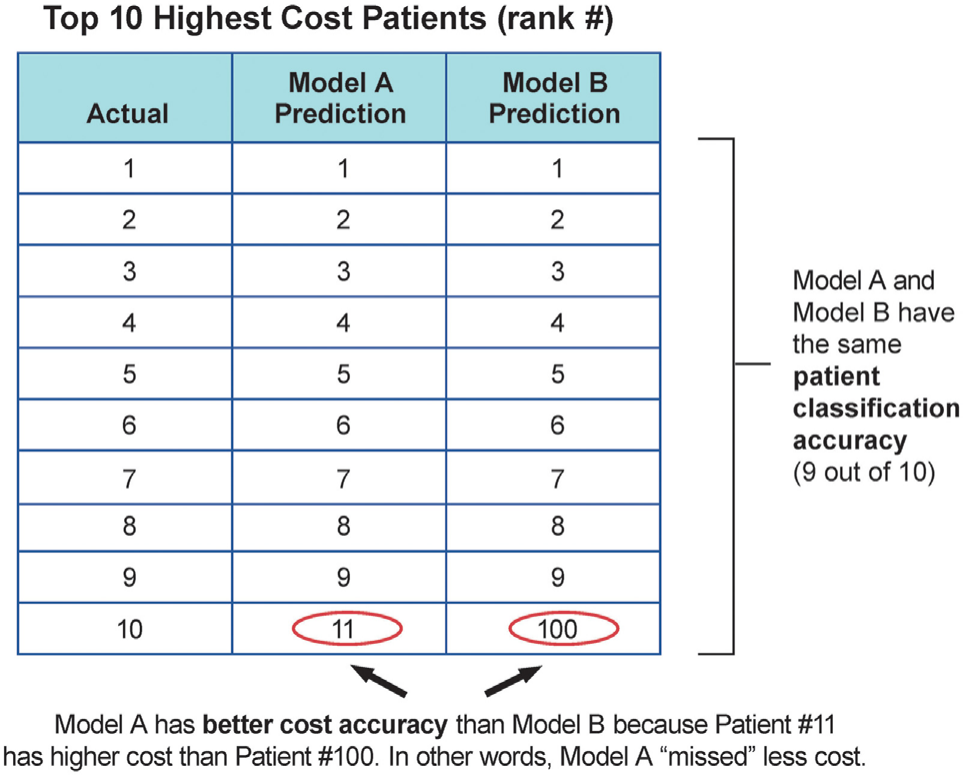
Troy example to demonstrate the value of cost accuracy, in addition to patient classification accuracy.

Models performance was evaluated with three main measures: the standard *R*^2^ measure in predictive regression, the classification accuracy of high-cost schizophrenia patients (see Eq. 1), and the cost accuracy of expenses resulting from high-cost schizophrenia patients (see Eq. 2) using different stratification thresholds. *R*^2^ measure reflects how close the data are to the fitted regression line. The patient classification accuracy (PCA) and cost accuracy (CA) are measures to represent the important prediction performance in this study. Ideally, the model should correctly identify all the high-cost patients with 100% classification accuracy so these patients can be well managed to alleviate future high expense. However, given the limited information and uncertainty, PCA and CA will be compromised. PCA reflects the need to capture the exact high-cost patients and allocate resources accordingly, and CA reflects the need to capture the relatively high-cost cohorts for population management. For example, with the same PCA, identifying high-cost patients with greater future expense should be prioritized over those with lesser future expense. Therefore, majority cost can be addressed using CA with the same PCA. Figure 2 illustrates the value of CA in addition to PCA in a troy example. Model A and B have the same PCA, but model A will provide better CA compared to model B. Model A provides more business value since it identifies patients with higher cost. According to the cost distribution in the test cohort, two thresholds were used for labeling high-cost patients: top 10% rank and 20% rank given the fact that the top 10% of patients consumed more than 50% of the overall cost (~$22 M out of $41 M per year), and the top 20% of patients consumed more than 70% of the overall cost (~$29 M out of $41 M per year). The performance measures were reported for each of the thresholds.

(1) $$\text{Patient Classification Accuracy}=\frac{\text{\# of correctly stratified high-cost patients}}{\text{\# of high-cost patients}}\times \text{100\%}$$

(2) $$\text{Cost Accuracy}=\frac{\text{actual cost of stratified high-cost patients}}{\text{actual cost of true high-cost patients}}\times \text{100\%}$$

## Results

### Model Performances and Cost Prediction

Table 2 compares the performance of the three stage models as well as the CMS-HCC model in the study evaluation cohort. The CMS-HCC model only achieved 0.09 *R*^2^, 27% PCA with 45% CA at top 10% high-cost patient cutoff, and 35% PCA with 57% CA at top 20% cutoff. With minimum demographic (age, gender) and total PMPM cost variables in OW, the Baseline Model using linear regression significantly improved the performance measures of *R*^2^ by 10%, PCA and CA by 13% at top 10% setting, and 15%, 9% at top 20% setting, compared to the CMS-HCC model. After adding the coverage, health-care utilization, antipsychotic medication usage, and detailed cost variables, the Enhanced Model further improved the performance measures of *R*^2^, PCA, and CA by ~2–5%, compared to Baseline Model. Whereas the CMS-HCC achieved an *R*^2^ of only 0.09 in the schizophrenic population, our Baseline Model (using age, gender, and total PMPM cost) achieved an *R*^2^ of 0.19, and adding coverage, health-care utilization, antipsychotic medication usage, and detailed costs increased the predictive value of this model (0.24 *R*^2^). Adding the sparse features of clinical condition proxies to the Enhanced Model, the Final Model further improved the PCA and CA performance by ~1–2%, using the top 500 features identified by Scalable Orthogonal Regression and random forests regression (*n* = 100).

**Table 2:** Performance measures in baseline model, enhanced model, and final model.

Measures	Baseline model	Enhanced model	Final model	CMS-HCC model
*R*^2^	0.19	0.24	0.24	0.09
PCA in top 10% setting, %	40	42	43	27
CA in top 10% setting, %	58	61	63	45
PCA in top 20% setting, %	50	52	53	35
CA in top 20% setting, %	66	68	69	57

*CA, cost accuracy; CMS-HCC, Centers for Medicare & Medicaid Services Hierarchical Condition Categories; PCA, patient classification accuracy*.

**Figure 3:**
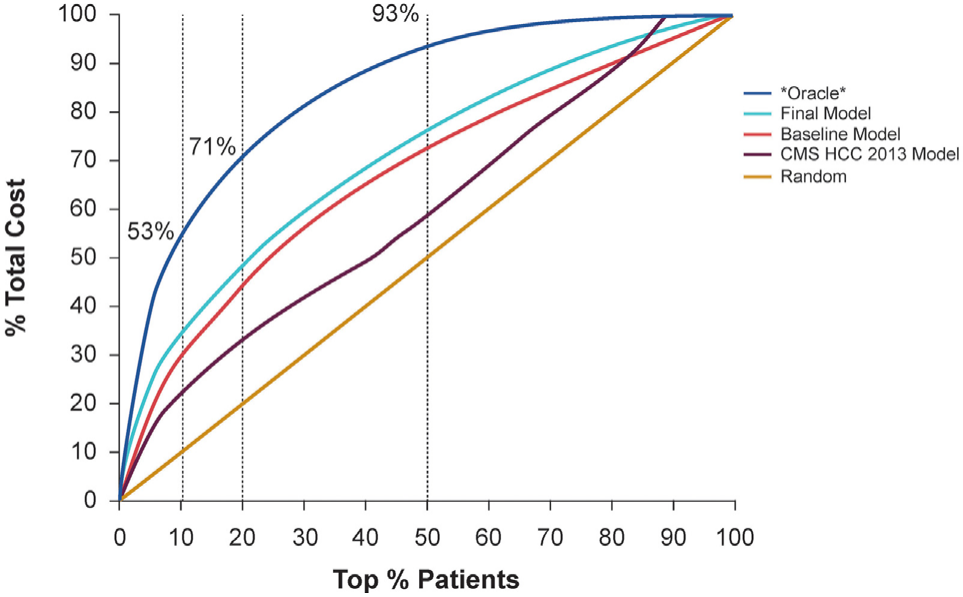
Demonstration of the cost captured by the baseline model and final model with different top % patient ranks, compared to random selection and *Oracle* selection that can predict the future with 100% accuracy.

Figure 3 demonstrates the cost captured by the Baseline Model and Final Model with different top % patient ranks, compared to random selection, the CMS-HCC model, and *Oracle* selection. *Oracle* selection reflects the actual cost distribution in the study evaluation cohort (i.e., with perfect knowledge of the future costs). In the study evaluation cohort, the top 10% of patients consumed 53.4% of total health expense; and the top 20% of patients consumed 71.0%. Furthermore, the top 50% of patients consumed almost all of the health expenses (93.4%).

Adding the detailed costs, utilization, and clinical condition variables, the Final Model captured 4% more total cost compared to the Baseline Model, using the top 10% patient rank as the high-cost threshold (improved from 29 to 33%), and captured 5% more total cost, using the top 20% patient rank as the high-cost threshold (improved from 44 to 49%). Considering the top 10% high-cost patient threshold, the Final Model identified 33% of total cost, compared with 22% for the CMS-HCC model and 29% for the Baseline Model.

### Predictive Feature Insights in the Final Model

Even though 500 features were involved in the Final Model, model performance reached its plateau at the top 20 variables. As expected, prior costs represented a majority (10/20), which included 12-month PMPM of total and detailed cost in standard utilization buckets (outpatient, inpatient, ED, office visit, and Rx), as well as the second half-year, Q4 PMPM of total cost, and Q4 PMPM of outpatient services. Twelve-month PMPM and the second half-year PMPM of total cost were the two leading predictive variables. Diabetes, chronic kidney disease, and anemias were critical clinical conditions listed in the top 20 variables. Age, office visit utilization, and antipsychotic medication adherence cumulative possession ratio, as well as procedure codes for anesthesia for salivary gland, and the 25-min office/outpatient visit from established patients were also among the top 20 variables.

## Discussion

Using advanced feature selection and supervised machine learning, and leveraging clinical costs and medication utilization data, we were able to identify more total cost, and more true high-cost patients with schizophrenia than the CMS-HCC model. In contrast to the CMS-HCC model, our model included not only demographics and ICD-9 diagnostic codes, but also added detailed cost, usage, procedure (CPT) code, and prescription data. As noted earlier, the CMS-HCC model was developed for a Medicare population and is being applied here to a broader population. Whereas the CMS-HCC achieved an *R*^2^ of only 0.09 in the schizophrenic population, our Baseline Model (using age, gender, and total PMPM cost), achieved an *R*^2^ of 0.19, and adding coverage, health-care utilization, antipsychotic medication usage, and detailed costs increased the predictive value of this model (0.24 *R*^2^). The results demonstrated the add-on value of that information in future cost prediction in addition to the baseline prior year cost. The observed low predictive ability (*R*^2^ less than 0.10) of the CMS-HCC in the schizophrenia population is consistent with previous work, which found that the HCC model performs relatively poorly in predicting costs in a population with behavioral or mental health issues (3, 8, 9). Our findings indicate that adding more specific variables improved the ability to predict total cost in a mental health population.

Our Final Model reached its plateau for prediction at the top 20 risk variables, which included costs (particularly 12-month PMP, and the second half-year PMPM of total cost), as well as the presence of critical clinical conditions including diabetes, chronic kidney disease, and anemia, and age, office visits, and antipsychotic medication adherence. The finding that comorbid conditions contribute to the costs is not surprising and is consistent with data showing that schizophrenia tends to co-occur with high rates of medical comorbidity, including diabetes, and to have higher comorbidity-related costs (17). Non-adherence to antipsychotic medication is also a key factor predicting risk of relapse, subsequent re-hospitalization, and higher hospitalization costs (18). Our findings underscore the importance of appropriate management of schizophrenic patients with comorbidities including diabetes, as well as ensuring that antipsychotic medication adherence is prioritized.

Even with the inclusion of more detailed features and variables than the CMS-HCC model, our analysis explains only up to 24% of the variance in costs. Using an expanded dataset, which includes details such as disease severity, patient cognitive functional status, social variables (e.g., degree of family/social support, homelessness, employment status), and duration of schizophrenia diagnosis, might help to improve the predictive power of the model.

Our current model is built on a commercial/self-insured coverage dataset and, therefore, may not apply to patients with schizophrenia on Medicaid, or other populations. Also, our model limited the analysis to patients who had been diagnosed with schizophrenia within the data collection period. Future studies should include an expanded population, including Medicaid and/or additional data sources such as criminal justice and social services data, as well as an expanded timeframe of data analysis. Future studies should also apply this approach for modeling risk and costs for patients with other mental/behavioral health diagnoses.

As we move toward a model for deployment, it would be helpful to be able to analyze relatively new patients entering the health-care dataset, as patients are increasingly becoming more mobile and seeking different insurance products and coverage (i.e., switching between public exchange and Medicaid, or switching insurance companies). Future investigations should be undertaken to refine our ability to predict risk for patients with a shorter enrollment history (e.g., only a few months of claims data).

## Conclusion

We found, using advanced feature selection and supervised machine learning methods, and leveraging detailed clinical and medication data, that there was an improvement in the ability to predict and identify high-cost patients with schizophrenia compared with the CMS-HCC model. Improving our ability to predict high-cost/high-risk patients with mental health issues including schizophrenia may provide support to health organizations to coordinate and deliver the right services to the most appropriate individuals.

## Prior Presentations

Wang Y, Iyengar V, Hu J, Kho D, Falconer E, Docherty J, Yuen G. Predictive Models to Identify Future High-Cost Schizophrenic Patients Using High Dimensional Administrative Data. Presented at AMCP Nexus 2016 Conference; October 3–6, 2016; National Harbor, Maryland.

## Author Contributions

All authors contributed to the conception or design of the work, or the acquisition, analysis, or interpretation of the data. All authors drafted the paper or revised it critically for important intellectual content, approved the final version for publication, and agree to be accountable for ensuring the integrity and accuracy of all aspects of the work.

## Conflict of Interest Statement

DK, EF, and JD are employees of ODH, Inc.; YW, VI, JH, and GY are or were employees of IBM T.J. Watson Research Center at the time the study was completed.
